# Prednisolone improves walking in Japanese Duchenne muscular dystrophy patients

**DOI:** 10.1007/s00415-013-7104-y

**Published:** 2013-09-22

**Authors:** Fumi Takeuchi, Naohiro Yonemoto, Harumasa Nakamura, Reiko Shimizu, Hirofumi Komaki, Madoka Mori-Yoshimura, Yukiko K. Hayashi, Ichizo Nishino, Mitsuru Kawai, En Kimura, Shin’ichi Takeda

**Affiliations:** 1 Department of Child Neurology, National Centre Hospital, National Centre of Neurology and Psychiatry, 4-1-1 Ogawahigashi, Kodaira, Tokyo 187-8551 Japan; 2 Translational Medical Centre, National Centre of Neurology and Psychiatry, 4-1-1, Ogawa-Higashi, Kodaira, Tokyo, 187-8551 Japan; 3 Department of Neurology, National Centre Hospital, National Centre of Neurology and Psychiatry, 4-1-1 Ogawahigashi, Kodaira, Tokyo, 187-8551 Japan; 4 Tokyo Women’s Medical University, 8-1, Kawada-cho, Shinjuku-ku, Tokyo, 162-8666 Japan; 5 Higashi-Saitama National Hospital, 4147, Kurohama, Hasuda, Saitama 349-0196 Japan; 6 Present Address: Department of Neurophysiology, Tokyo Medical University, 6-1-1 Shinjuku, Shinjuku-ku, Tokyo, 160-8402 Japan

**Keywords:** Duchenne muscular dystrophy, Prednisolone, Walking, National registry, Natural history

## Abstract

We evaluated the long-term efficacy of prednisolone (PSL) therapy for prolonging ambulation in Japanese patients with genetically confirmed Duchenne muscular dystrophy (DMD). There were clinical trials have shown a short-term positive effect of high-dose and daily PSL on ambulation, whereas a few study showed a long-term effect. Especially in Japan, “real-life” observation was lacking. We utilized the national registry of muscular dystrophy in Japan for our retrospective study. We compared the age at loss of ambulation (LOA) between patients in PSL group and those in without-PSL group. Out of 791 patients’ in the Remudy DMD/BMD registry from July 2009 to June 2012, 560 were matched with inclusion criteria. Of the 560, all were genetically confirmed DMD patients, 245 (43.8 %) of whom were treated with PSL and 315 (56.2 %) without PSL. There was no difference between the two groups regarding their mutational profile. The age at LOA was significantly greater (11 month on average) in the PSL group than in the without-PSL group (median, 132 vs. 121 months; *p* = 0.0002). Although strictly controlled clinical trials have shown that corticosteroid therapies achieved a marked improvement in ambulation, discontinuation of the drug due to intolerable side effects led to exclusion of clinical trial participants, which is considered as unavoidable. In our study, patients were not excluded from the PSL group, even if they discontinued the medication shortly after starting it. The results of our study may provide evidence to formulate recommendations and provide a basis for realistic expectations for PSL treatment of DMD patients in Japan, even there are certain limitations due to the retrospectively captured data in the registry.

## Introduction

Duchenne muscular dystrophy (DMD) is a rare disease linked to the X-chromosome that affects 1 in 5,000–6,000 newborn males [1]. The disorder follows a progressive course of muscle weakness and also involves cardiac and respiratory muscles. DMD is caused by mutations in the *DMD* gene, which results in severe reduction or complete elimination of the dystrophin protein. Although the molecular origins of DMD have been known for several years, there is still no curative treatment for the disease. It has been nearly four decades since the potential benefits of glucocorticoids (GCs) for DMD were first reported by Drachman et al. [2]. In the years since, several randomised controlled trials (RCTs) have concluded that GCs increase short-term muscle strength and improve muscle function (from 6 months to 2 years) [3–7] with frequent but not severe adverse effects [6]. In contrast, the long-term benefits and adverse events of GCs have not yet been assessed by an RCT [4], although non-RCTs have suggested functional benefits for over 5 years in some GC-treated patients [8–17]. However, these studies were conducted in small numbers of patients. While PSL has been available for DMD patients since 1990s, there has been very little literature regarding the regimens of PSL for DMD in Japan. Some Japanese experts have a vague idea that the adequate dose could be lower than the one recommended (0.75 mg/kg/day) based on their expert experiences. Deflazacort has not been available yet in Japan [18].We used a large national registry of DMD patients in Japan to conduct a retrospective study on the long-term clinical efficacy of PSL therapy for maintenance of unassisted ambulation in DMD patients.

## Methods

**Fig. 1:**
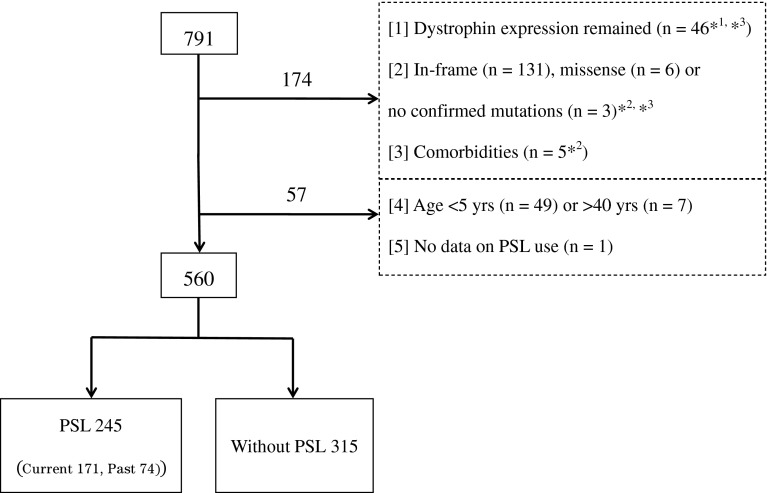
Selection of DMD patients from the Remudy database for this study. *1: These 46 patients included out-of-frame mutations (*n* = 7) and nonsense mutations (*n* = 7). *2: Three patients met (2) and (3) of our exclusion criteria. *3: Twenty-three patients met (1) and (2) of our exclusion criteria. *DMD* Duchenne muscular dystrophy, *PSL* prednisolone, *Remudy* Registry of Muscular Dystrophy

In 2009, we developed a national registry of Japanese DMD/BMD patients (Remudy) in collaboration with the Translational Research in Europe-Assessment and Treatment of Neuromuscular Diseases (TREAT-NMD) Network of Excellence [19, 20]. The Remudy database includes clinical and molecular genetic data as well as all required items for the TREAT-NMD global patient registry. The database includes male Japanese DMD/BMD patients throughout Japan whose genetic status has been confirmed by genetic analysis. The registry data includes age at registration, birth date, area of residence, features of the muscle biopsy, genomic mutations, complicating diseases, PSL use (present use, past use or never), present functional mobility, age at LOA, cardiac function, respiratory function, spinal surgery, serum CK level, family history of DMD etc., but does not includes PSL regimes (dose, age at commencement and duration), side effects of PSL or physiotherapy. All these data were confirmed by three molecular and two clinical curators in Remudy. In this study, we used the registry data compiled from July 2009 to June, 2012 to compare the clinical course of DMD between patients with and without PSL therapy. Patients were excluded for any of the following reasons: (1) dystrophin expression remained on muscle biopsy by immunohistochemistry test; (2) in-frame, missense or unconfirmed mutation of *DMD gene* by mutation screenings; (3) comorbidities, such as adrenal hypoplasia or nephrotic syndrome; (4) current age ≤5 years or ≥40 years (because PSL therapy for DMD was not common before the 1990s) or (5) missing data on PSL use (Fig. 1). We compared the age at LOA between PSL group of patients, which was comprised of both current and past PSL-treated patients, and without-PSL group, which was comprised of patients who had never been treated with PSL (steroid naïve). The primary outcome measure was ‘independent walking’ defined as ‘unsupported walking indoors’ [11], which is one of the standardized items in the TREAT-NMD global registry format. Because LOA was not well defined in several previous studies, there is no clear consensus on the definition of LOA [11]. The Kaplan–Meier method was used to analyse the age at LOA, and the log-rank test was used to compare differences between PSL group and without-PSL group. We used age at LOA as a primary outcome because the database did not contain information on the initiation or duration of PSL treatment [21]. We set 5 years as the start time for PSL therapy. We used the Cox regression model to perform univariate and multivariate analyses to assess the effect of PSL. A covariate selected for adjustment was area of residence because the registrants varied in number and frequency of PSL treatment among 6 geographical areas. In addition, we considered family history of DMD as another covariate for adjustment because it might have influenced the patients’ decisions to accept PSL treatment. We calculated hazard ratios (HRs) and their 95 % confidence intervals (CIs). Statistical significance was defined as a two-sided *p* value <0.05. The software, SAS version 9.2 (SAS Institute Inc., Cary, NC, USA), was used to perform all statistical analyses. We also searched the PubMed database, reviewed related studies on the long-term effect of GCs on preservation of ambulation, and compared these previous results to those reported in the present study.

## Results

### Demographics

Of the 791 patients (from 147 hospitals, with 228 doctors’ cooperation) in the Remudy database, 174 were excluded because they met at least 1 of the exclusion criteria, and dystrophin expression remained on muscle biopsy tissue was observed in 46 patients. One hundred and forty patients were excluded by *DMD gene* mutation screening, 131 had in-frame mutations, 6 had missense mutations and 3 did not show mutations detectable with standard methods (MLPA, exonic sequencing). Five had comorbid diseases, such as nephrotic syndrome and adrenodysplasia. We also excluded 57 patients because 49 were <5 years old, 7 were ≥40 years old and there was missing data on the use or non-use of PSL for 1 patient. After removing patients who fulfilled at least 1 exclusion criterion, the final group for analysis included 560 genetically confirmed DMD patients (Fig. 1).

Baseline characteristics are presented in Table 1. The mean current age of the 560 patients was 15.4 years, and the median current age was 14.0 years (interquartile range, 9–20 years). Of the 560 patients included, 245 (43.8 %) were in PSL group, and 315 (56.2 %) were in without-PSL group. The PSL group included 74 patients who had been treated with PSL in the past and 171 patients were currently on PSL (Fig. 1). Table 1 also presents the features of the *DMD gene* mutations in the PSL group and without-PSL group. Mutations included exon deletions or exon duplications (PSL patients: 183/245, 74.7 %; without-PSL patients: 230/315, 73.0 %); small frame shifts, deletions or insertions (PSL: 21/245, 8.6 %; without-PSL: 26/315, 8.3 %) and nonsense mutations (PSL: 29/245, 11.8 %; without-PSL: 41/315, 13.0 %). There was no difference in the mutation type distribution between the 2 groups. On the other hand, the geographic distribution of the 2 groups was significantly different, between 12 and 63 % of patients received PSL. We also presented distribution of the year-of-birth (per decade) in both PSL group and without-PSL group. The patients (PSL group and without-PSL group) were distributed in 2001–2010 (87/245, 35.5 %; 106/315, 33.7 %), 1991–2000 (131/245, 53.5 %; 120/315, 38.1 %), 1981–1990 (24/245, 9.8 %; 60/315, 19.0 %) and 1971–1980 (3/245, 1.2 %; 29/315, 9.2 %) respectively.

**Table 1:** Patient characteristics

		PSL		Without-PSL		Total
	Total	*n*	%	*n*	%	*n*
		245	100.0	315	100.0	560
Mutation	Exon del/dup	183	74.7	230	73.0	413
	Frame shift or small del/ins	21	8.6	26	8.3	47
	Nonsense	29	11.8	41	13.0	70
	Others	12	4.9	18	5.7	30
Family history	Yes	60	24.9	110	34.9	170
	No	185	75.1	205	65.1	390
Region	Hokkaido and Tohoku	17	9.6	13	4.1	30
	Kanto	148	60.4	87	27.6	235
	Chubu and Tokai	33	13.5	73	23.2	106
	Kansai	25	10.2	62	19.7	87
	Chugoku and Shikoku	14	5.7	23	7.3	37
	Kyusyu and Okinawa	8	3.3	57	18.1	65
Year of birth	2001–2010	87	35.5	106	33.7	193
	1991–2000	131	53.5	120	38.1	251
	1981–1990	24	9.8	60	19.0	84
	1971–1980	3	1.2	29	9.2	32

*PSL* prednisolone, *del* deletion, *dup* duplication, *ins* insertion

### Outcome

**Fig. 2:**
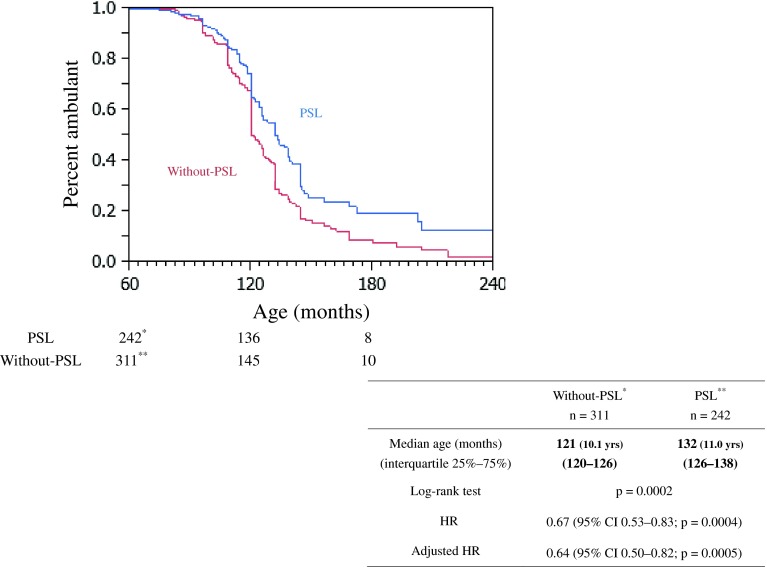
Time to loss of ambulation in the PSL group and without-PSL group determined by the Kaplan–Meier method. *Three patients in the PSL group and. **Four patients in the without-PSL group were excluded because their ambulation status was unknown. The PSL group had 242, 136 and 8 ambulant patients at 60, 120 and 180 months of age, respectively. The without-PSL group had 311, 145 and 10 ambulant patients at 60, 120 and 180 months of age, respectively. *PSL* prednisolone, *HR* hazard ratio

Of the 560 patients, we excluded three patients from the PSL group and four from the without-PSL group because ambulation status was unknown. Finally, 553 patients, 242 in the PSL group and 311 in without-PSL group were included in the analysis. LOA was reported in 190 of the 311 patients in without-PSL group and 123 of the 242 patients in PSL group. The median age at LOA was 121 months (10.1 years, interquartile range: 120–126 months) for the patients in without-PSL group and 132 months (11.0 years, interquartile range: 126–138 months) in PSL group (Fig. 2). The HR for without-PSL group versus PSL group was 0.67 (95 %CI: 0.53–0.83, *p* = 0.0004), and the adjusted HR was 0.64 (95 %CI: 0.50–0.82, *p* = 0.0005).

## Discussion

To our knowledge, this is one of the largest studies world-wide on the long-term effects of PSL therapy on prolongation of independent walking ability in DMD and also the first study in Japan (Table 2). Historically, most DMD patients lose the ability to walk between 9 and 11 years of age [22], but recent improvements in care may have increased the age at LOA slightly even without the administration of steroids. In our study, the median age at LOA in patients who were never treated with PSL (without-PSL group) was 10.1 years. In a recent natural history study of 371 DMD boys, those on any steroid regimen for >6 months walked significantly longer (median age at LOA 12.0 years) than those on any regimen for <6 months or never on steroid (10.0 years) [23], which is quite similar to those without-PSL in our study. According to previous studies, patients receiving GC treatment were able to ambulate 2–5 years longer than those not treated with GCs [8, 23]. In the current study, patients treated with PSL were able to ambulate 11 months (0.9 years) longer on average than those without PSL, and the extension was relatively modest as compared to previous studies (Table 2). This may be due to one or several of the following factors: differences in ethnic origin of the treated population: small size of some of the previous studies: differences in the clinical definitions of DMD, different definitions of ambulation, variations in PSL regimens, and most importantly duration of treatment. First, previous studies only have been conducted in small numbers of patients (129 patients at most [12]), whereas the sample size in our study was 560 patients. On the other hand, Ricotti et al. [24] performed a prospective observational study in 360 patients, but their study did not compare a GC-treated group to a non-treated group. Second, the genetic and molecular criteria used to define DMD have varied between studies (Table 2). In the Leiden *DMD* mutation database, 9 % of the mutations did not follow the reading-frame rule [25]. A diagnosis based on a purely molecular genetic approach may not accurately distinguish DMD from Becker muscular dystrophy and milder dystrophinopathies, especially in young children with no family history of DMD. In these patients, a muscle biopsy can help verify dystrophin expression to confirm the existence and severity of a functional mutation in the *DMD gene* [26]. Using *DMD gene* analysis only, previous studies may have included subjects with a milder phenotype (residual dystrophin expression) with longer prolongation of independent ambulation regardless of GC treatment history. To improve the precision of diagnosis in our study, we excluded all patients who had any residual dystrophin expression in muscle tissue. However, 303 patients in our study were diagnosed as having DMD only based on *DMD gene* analysis. Of the 303 patients, 125 (28 treated in the past, 97 currently being treated) were in PSL group (50.0 % of 250), and 178 were in without-PSL group (56.5 % of 315). Therefore, some patients with milder phenotype may have been included in both groups. Third, PSL regimes (dose, age at commencement and duration) in our study may possibly have differed from those in related studies. A few previous studies only enrolled patients treated with GC for >1 [4] or >2 [8] years before LOA. Strictly controlled clinical trials have shown a more marked improvement in ambulation. However, discontinuation of the drug due to intolerable side effects leads to exclusion of clinical trial participants, while in our study patients were not excluded from PSL group, even if they discontinued the medication shortly after starting it. The American Academy of Neurology [27] and the Cochrane review [6] evaluated all RCTs on the use of GCs in DMD and concluded that PSL administered at 0.75 mg/kg/day was effective. However, a broadly accepted GC dose–response relationship has not been defined [6]. Therefore, a large-scale prospective study using strict criteria has been started very recently to determine the optimal regime in DMD (FOR-DMD) [28].

**Table 2:** Related studies on long-term effect of GC on preservation of ambulation

	Study design	Treated Non-treated Numbers	Criteria			Definition of loss of ambulation	Loss of ambulation	
			DMD gene analysis		Muscle biopsy		Treated Control Median age (Years)	Prolonged ambulation, (Years)
Our study	Ret	245 (P) 315	Exclude in-frame, missense, not confirmed mutations		Exclude residual Dys	Unable to walk, unsupported indoors	11.0 10.1	0.9
Ricotti [24]	Pro	360 (Pi191, Pd169) –	Include DMD mutation	or both	Include Dys (−)	NorthStar Ambulatory Assessment	Pi12.0, Pd14.5 –	2.5^a^
Merlini [13]	Pro	4 (P + D) 3	Out of frame in 3 patients		Include Dys (−)	10 m and 6 min walk	16–18^b^ ―	–
Bach [15]	Ret	17 (P16, D1) 117	Unknown		Include Dys (−)	Wheelchair dependence	10.8* 9.7*	1.1
Straathof [11]	Ret	35 (Pi) 0	Unknown		Unknown	Unable to walk, unsupported indoors	10.8 –	–
Houde [10]	Ret	37 (D) 42	Include deletions		Include Dys (−)	Can no longer walk even with help	11.5* 9.6*	1.9
King [12]	Ret	91 (P36 D25) 68	Exclude BMD-like mutation and phenotype		Unknown	Functional walking without orthoses or any assistive device	12.5* 9.2*	3.3
Pradhan [14]	Pro	15 (P) 19	Include deletions		Unknown	Chair-bound stage	14.0* 11.0*	3.0
Biggar [9]	Ret	40 (D) 34	Include deletions	and or	Include consistent with DMD	Unable to walk independently	–^c^ 9.8*	3–5^c^
Balaban [8]	Ret	30 (P18, D12) 19	Unknown		Unknown	Unable to walk 30 feet on a level floor	P10.6–12.4*, D10.9–12.9* 8.9–9.9*	–
Yilmaz [16]	Pro	66 (P) 22	Unknown		Unknown	Loss of independent walking ability	10.0* 8.6*	1.4

*Pro* Prospective study, *Ret* Retrospective study, *D* Deflazacort, *P* Prednisolone (Prednisone); *Pi* Intermittent Prednisolone, *Pd* daily prednisolone, *Dys* dystrophin expression

^a^Comparison between Pd and Pi

^b^4 Treated patients (age 16–18) were fully ambulant, able to fast walk 10 m, and to perform the 6MWT; three of them were still able to climb stairs

^c^ All treated boys could walk 10 m at 10 years of age, 25 (81 %) of 31 at 12 years, 13 (76 %) of 17 at 15 years and two of six boys walked independently at 18 years of age

* Mean age, years

Our study is limited because all data is retrospectively captured by the registry. The registry items does not include detailed information of PSL regimes (dose, age at commencement and duration), physiotherapy, or other additive treatments such as creatine [29, 30]. Although we adjusted for family history and area of residence in the multivariate analysis, there was still some possibility of residual confounding between the two groups, such as progression of the attitude of “the standards of DMD care” by the decades. There was no item regarding the side effects of long-term PSL administration. Thus, we did not conclude that the benefits of PSL treatment outweigh the risks. The most frequent adverse effect of long-term GC treatment was a reduction in a patient’s height [6]. Weight gain was the second most frequent adverse event and the reason most often cited for discontinuing treatment [17]. However, weight gain in GC-treated DMD patients was a multifactorial effect due to pharmacological effects of GC and patients immobility, because weight gain generally was more pronounced in non-ambulatory patients [31].

However, our observational study showed actual clinical setting of GCs therapy in Japan (“real life” data). The result of our study could provide evidence to formulate recommendations and base realistic expectations for steroid treatment of DMD patients in Japan. The residential variation in PSL use, depending on the geographical region of Japan, probably due to differing practices among hospitals and doctors, suggested that PSL therapy for the DMD patients had not been standardised in Japan [18]. Clinical practice guidelines for DMD in Japan will be published by the end of 2013. (http://www.neurology-jp.org/link/index.html, accessed August 12th, 2013). Finally, our data presents the first large outcome study of DMD patients in an Asian country. Recently, well conducted natural history studies for DMD have been reported from Europe and North American countries [23, 32]. Considering feasibility of global clinical trials for DMD, it appears relevant to obtain natural history data in non-western DMD patient populations. This study could add important information of the “real life” of DMD patients.
